# Long-acting muscarinic antagonists for the treatment of asthma in children—a new kid in town

**DOI:** 10.1007/s40629-018-0066-y

**Published:** 2018-04-26

**Authors:** Eckard Hamelmann

**Affiliations:** 1 Kinderzentrum Bethel, Evangelisches Klinikum Bethel, Grenzweg 10, 33617 Bielefeld, Germany; 2 Allergy Center of the Ruhr University, Bochum, Germany

**Keywords:** Children, Asthma, Treatment, Tiotropium

## Abstract

**Background:**

Asthma is the most prevalent chronic airway disease observed in children and adolescents, yet the variety of treatment options available for this age group is limited. With many factors influencing therapeutic efficacy including patient knowledge, adherence, and therapy choice as well as delivery device, it is important to have more options to tailor to individual patient needs.

**Methods:**

This article is an overview of recent scientific articles using a systematic literature search in PubMed and specialist databases.

**Results:**

Tiotropium is the first long-acting muscarinic antagonist to be licensed for treatment of asthma and has been demonstrated to be an effective add-on therapy across all age groups. Its therapeutic success in clinical trials resulted in Food and Drug Administration and the European Medicines Agency approval for asthma treatment in people over the age of 6 years in the US and EU.

**Conclusion:**

Further studies into the use of tiotropium, especially in younger children, could be of interest for future treatment decisions.

## Introduction

**Fig. 1:**
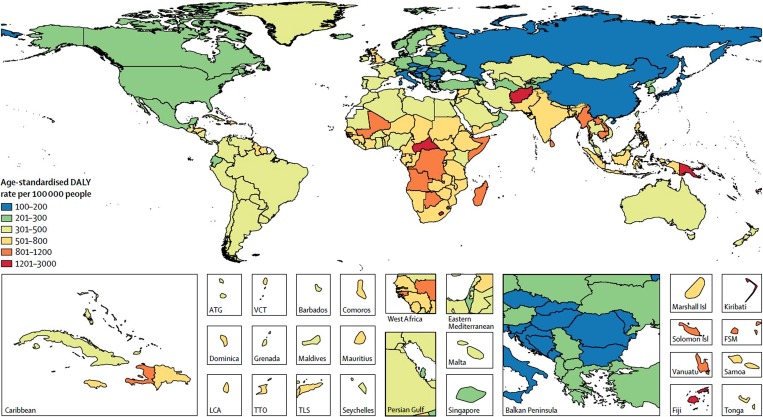
Age-standardised DALY rate per 100,000 people by country in 2015. Age-standardised DALY rate per 100,000 people due to asthma, by country, both sexes, 2015. *DALYS* disability-adjusted life years, *ATG* Antigua and Barbuda, *FSM* Federated States of Micronesia, *Isl* Islands, *LCA* Saint Lucia, *TLS* Timor-Leste, *TTO* Trinidad and Tobago, *VCT* Saint Vincent and the Grenadines. (Reproduced from [3] with permission from the Lancet)

Asthma is a disease characterised by chronic airway inflammation and is estimated to affect over 300 million people worldwide [1, 2]. The total burden of the disease, measured by disability-adjusted life years, ranks asthma as the second most important respiratory disease (Fig. 1; [1, 3]). Of note, asthma is one of the most frequent chronic diseases observed in children and adolescents, and the most common airway disease in this age group [2]. Globally, approximately 14% of children experience asthma symptoms such as wheezing, shortness of breath and chest tightness, and although childhood deaths from asthma are rare, they are still measurable, ranging between 0.0–0.7 per 100,000 children [1]. Indeed, positive associations between poor asthma control, exacerbations, hospital admissions and mortality have been consistently reported [4]. These factors may be used to identify patients at high risk of future exacerbations and loss of lung function, and may help to target appropriate preventative measures.

In this review, we discuss the importance of improving the management of paediatric asthma, including the unmet need for alternative treatments. In this context, we examine the potential use of the long-acting muscarinic antagonist (LAMA) tiotropium as an add-on therapy for asthma management in children and adolescents aged between 6–17 years.

## Treatment and management

Irrespective of age, the main objectives of asthma management are to achieve symptom control while minimising the future risk of exacerbations, fixed airflow limitation and side effects of treatment [2]. The Global Initiative for Asthma (GINA) and the Guideline of the German Respiratory Society [5] recommend that therapy in school-age children and adolescents should be stepped up if control is not achieved with low-dose inhaled corticosteroids (ICS) and on-demand short-acting inhaled beta2-agonists (SABAs), through the addition of either a long-acting beta2-adrenoreceptor agonist (LABA) or a leukotriene receptor antagonist (LTRA), or by further increasing the prescribed dose of ICS [2]. If the disease is still not controlled, the addition of tiotropium is a recommended step for adolescent patients at GINA Step 4. However, it is important to note that country-specific guidelines for the management of asthma in children and adolescents may recommend different treatment regimens [2, 5–7]; for example, the LAMA tiotropium has recently been approved for the treatment of asthma in people aged ≥6 years in the United States and the European Union [8, 9].

The use of pharmacological treatment is also fundamental for the management of asthma in children, and achieving the right balance between drug safety and efficacy is especially paramount in this age group. ICS are established as the most effective anti-inflammatory treatment and remain the mainstay therapy for all types of asthma. Regular use of ICS is known to improve asthma control, reduce frequency and severity of symptoms, decrease the use of SABAs for symptom relief, improve quality of life and decrease the risk of acute asthma exacerbations [10]. However, the continued use of ICS in children has been found to significantly affect their adult height [11]. In children aged 5–13 years, a height deficit was observed 1 to 2 years after treatment initiation with ICS that persisted into adulthood [11]. The regular reassessment of asthma control, growth rate monitoring and step-down ICS therapy in young patients with controlled disease is, therefore, mandatory, and should decrease side effects without affecting treatment efficacy and safety.

The safety profile of SABAs—the most commonly used reliever medication for paediatric patients—is generally favourable, with reports of mild side effects such as tachycardia, dizziness and jitteriness [12]. LTRAs—which are a potential alternative first-line therapy, either as monotherapy or in combination with ICS—reduce exacerbation rates and use of SABAs in children with asthma of varying severity [13]. They are also generally well tolerated, except for minor side effects such as headaches and gastrointestinal upset [12].

Addition of LABAs to low-dose ICS therapy for children >6 years with uncontrolled asthma is more likely to provide a positive therapeutic response and improved disease control than either ICS or LTRA step-up [14]. However, incorrect use of LABAs (especially as monotherapy) has been associated with increased rates of hospitalisation [15] and asthma-related deaths [16], and is linked to the occurrence of severe adverse events [12]. LABAs have also been found to lose efficacy over time due to tachyphylaxis [17]. There is therefore a need for additional well-tolerated add-on options.

## Adherence and education

Despite currently available treatments, complete disease control is still not achieved in the majority of patients. Indeed, management of asthma is a multifactorial process, and prescription of the most appropriate treatment strategy is only one aspect. Disease management is particularly challenging in young children and adolescents due to underdiagnosis, while earlier diagnosis—and thus treatment—of asthma may improve the long-term prognosis [18]. The difficulties faced in this age group include underpresentation of symptoms by patients and incorrect diagnosis by healthcare practitioners (HCPs; [19]).

Once a patient is diagnosed and therapy is initiated, ensuring that the treatment strategy is adhered to and that it is continuously monitored is also extremely important for the management of the disease. Poor medication adherence is a widespread problem: it increases morbidity and medical complications, contributes to the decrease in quality of life and increases the burden on the healthcare system [18, 20].

Nonadherence includes conscious nonadherence, with patients unwilling to take medication as prescribed and not attending medical appointments, and subconscious nonadherence, such as incorrect inhalation device-use or forgetting to take medication as prescribed [21]. Asthma regimens often require planning and forethought, including filling prescriptions and taking treatment at the correct time. Inadequate instructions, or a regimen that is too complex or time-consuming are important considerations when assessing adherence. Accurate inhalation technique training is therefore critical for treatment success [22]. Recent studies have found medication adherence to be negatively associated with age [23]. As children mature, parents/caregivers may decrease supervision, increasing the child’s responsibility for their own asthma management. Younger children may not remember to take medications, whereas adolescents may choose not to take treatment in certain situations to avoid embarrassment in front of their peers [24]. If nonadherence goes undetected and the disease becomes uncontrolled, an unnecessary increase in the dose of medication or an additional treatment may be prescribed to try to achieve disease control, increasing the cost and complexity of the regimen [21].

A basic understanding of the disease and information about the treatment patients are prescribed should increase their adherence. Lack of understanding of the disease amongst children and/or their parents may result in incorrect use of an inhaler device, causing ineffective drug delivery to the lungs and a reduction of medication efficacy. Altered patient/carer perception of treatment success can cause them to change treatment strategy or reduce therapy without consulting an HCP, placing the patient at higher risk of exacerbations. Adherence improves when physicians inform and collaborate with their patients to ensure complete and correct understanding of asthma and the necessary treatment [25]. Clear and open patient–physician communication addressing patient-specific concerns, engaging patients in the management of their disease, ensuring family members are educated and able to support the patient and the necessary therapy regimes, and regular follow-up appointments with the treating physician are important components for success. Only with honest, clear and open communication can both patient/carer and physician recognise any symptom changes and adjust treatment according to guidelines, or switch to any available alternative medication options.

Satisfaction with inhaler devices also correlates with improved adherence and clinical outcomes [26]. Four classes of inhaler are currently available: pressurised metered-dose inhalers (pMDIs), which require coordination between actuation and inhalation; dry powder inhalers (DPIs), which need strong inspiration; soft mist inhalers (SMIs), which are propellant-free and powered by a tensioned spring; and nebulisers [27]. Some studies suggest that specific inhaler types may be associated with better adherence, although further investigation is needed to confirm this [26]. In addition, different inhalers require different handling techniques, which can introduce complexity, and may confuse patients and result in nonadherence [28].

## Tiotropium—the new kid on the block

Regular reviews to ensure that patients are prescribed the most appropriate therapy option are key for the management of asthma. GINA suggests that patients’ response to treatment be regularly reviewed based on symptoms, exacerbations, side effects, lung function and patient satisfaction with treatment [2].

The current guideline-based drug therapy for childhood asthma is primarily based on extrapolated data from studies carried out in adults. There are very few products that are licensed for paediatric use in asthma, with limited research for mild-to-moderate disease or younger patients, and more focus on severe asthma and adolescents/children >12 years.

Tiotropium is a LAMA that has been approved for the treatment of chronic obstructive pulmonary disease for over a decade [29]. Tiotropium reduces inflammation [30] and increases bronchodilation through inhibition of M3 receptors at the smooth muscle [31]. The positive findings from multiple recent phase II and III clinical trials in adult patients with symptomatic asthma resulted in tiotropium becoming the first LAMA approved for asthma therapy [29].

More recently, tiotropium has been demonstrated to be an effective add-on therapy for patients with asthma across all age groups. Phase II trials in children and adolescents confirmed safety and tolerability profiles that were similar to those observed in adults [32, 33]. Following the success of these trials, five phase III trials examined the efficacy and safety of tiotropium as add-on therapy in children and adolescents with symptomatic moderate-to-severe asthma (Table 1). In the 48-week RubaTinA-asthma® study, once-daily tiotropium significantly improved lung function in adolescent (12- to 17-year-olds) patients with symptomatic moderate asthma [34]. In patients from the same age group with more severe asthma, an overall positive trend in lung function and asthma control was observed in the 12-week PensieTinA-asthma® study [35]. In addition, two studies conducted in children aged 6–11 years with symptomatic severe asthma (VivaTinA-asthma®; [36]) and symptomatic moderate asthma (CanoTinA-asthma®; [37]) demonstrated improved peak forced expiratory volume in 1 s 3 h post-dose compared with placebo, and reported that tiotropium was well tolerated as an add-on therapy to ICS. A small safety study in preschool children aged 1–5 years with persistent asthmatic symptoms (NinoTinA-asthma®) reported that the tolerability of tiotropium was similar to that of placebo add-on therapy to ICS [38]. Patients treated with tiotropium reported significantly fewer adverse events related to asthma exacerbations and asthma worsening compared to placebo, indicating tiotropium’s potential to reduce asthma exacerbation risk compared with placebo, although further well-powered trials are required to further assess this [38]. The side effect profile of tiotropium in children and adolescents is comparable to that observed in adult patients with asthma. The most commonly reported side effects include sore throat, headache, bronchitis and sinus infection and, overall, in large clinical trials the proportion of patients reporting adverse events with tiotropium was comparable with placebo [34–41].

**Table 1:** Key results from phase III studies with tiotropium in children and adolescents with asthma

Age group	Study	Patients (*n*)	Treatment (weeks)	Lung function	Difference from placebo (mL, 95% CI)			
					5 µg tiotropium		2.5 µg tiotropium	
6- to 11-year-olds	VivaTinA-asthma [36]	401	12	Peak FEV_1_ (Week 12)	139 (75.203)	*p* < 0.001	35 (−28.99)	*p* = 0.27
				Trough FEV_1_ (Week 12)	87 (19.154)	*p* = 0.01	18 (−48.85)	*p* = 0.59
	CanoTinA-asthma [37]	401	48	Peak FEV_1_ (Week 24)	164 (103.225)	*p* < 0.001	170 (108.231)	*p* < 0.001
				Trough FEV_1_ (Week 24)	118 (48.188)	*p* = 0.001	116 (46.186)	*p* = 0.001
12–17-year-olds	PensieTinA-asthma [35]	392	12	Peak FEV_1_ (Week 12)	90 (−19.198)	*p* = 0.14	111 (2.220)	*p* = 0.046
				Trough FEV_1_ (Week 12)	54 (−61.168)	*p* = 0.361	115 (0.231)	*p* = 0.051
	RubaTinA-asthma [34]	398	48	Peak FEV_1_ (Week 24)	174 (76.272)	*p* < 0.001	134 (34.234)	*p* < 0.01
				Trough FEV_1_ (Week 24)	117 (10.223)	*p* = 0.03	84 (−25.194)	*p* = 0.13

*CI* confidence interval, *FEV*_*1*_ forced expiratory volume in 1 s, *NS* not significant

**Fig. 2:**
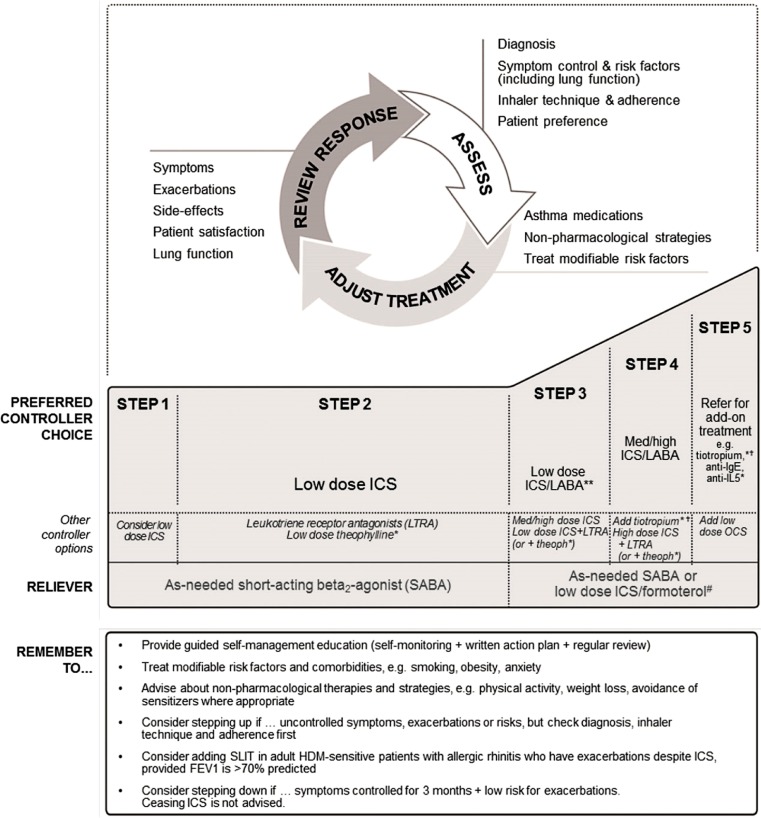
GINA recommended step-wise approach for asthma symptom control and minimizing future risk. Low, medium, and high doses of ICS differ between adults, adolescents, and children 6–11 years. *ICS* Inhaled corticosteroids, *LABA* long-acting beta-agonist, *med* medium dose, *OCS* oral corticosteroids. *Not for children <12 years. **For children 6–11 years, the preferred Step 3 treatment is medium dose ICS. *#*Low dose ICS/formoterol is the reliever medication for patients prescribed low dose budesonide/formoterol or low dose beclomethasone/formoterol maintenance and reliever therapy. *†*Tiotropium by mist inhaler is an add-on treatment for patients with a history of exacerbations, it is not indicated in children <12 years. (Reproduced from reference [2] with permission from GINA)

The GINA report now recommends tiotropium as an add-on option for adolescents (aged ≥12 years) with a history of exacerbations [2]. More recently, the Federal Drug Administration and the European Medicines Agency approved the use of tiotropium as maintenance therapy for children with asthma aged >6 years after the observed success of the tiotropium paediatric clinical trial programme (Fig. 2; [8, 9]).

For any therapy to be approved for paediatric use, evidence is required that it has been sufficiently evaluated for the proposed indication. However, the International Council for Harmonisation suggests that extrapolation from adult data is possible when the disease has a similar course in both adults and children, and when the therapeutic outcome is likely to be similar [42]. The tiotropium paediatric trials—CanoTinA-asthma® [37], VivaTinA-asthma® [36], RubaTinA-asthma® [35] and PensieTinA-asthma® [34]—found a comparable degree of improvement in both peak and trough FEV_1_ to that observed in adults (Table 1; [43]). The available data are limited due to ethical considerations and the short duration of trials in patients with symptomatic severe asthma.

Data from the tiotropium paediatric clinical trial programme support further extrapolation of efficacy and safety data from larger adult trials to children and adolescents, thereby avoiding unnecessary large paediatric studies. Subgroup analyses of previous trials with tiotropium in adults demonstrated that immunoglobulin E levels, blood eosinophil counts and type or dosage of maintenance therapy did not affect tiotropium efficacy in patients with symptomatic asthma [44, 45]. More recently, post hoc analysis in adult patients with asthma demonstrated an improvement in lung function upon addition of tiotropium to maintenance therapy across GINA Steps 2–5 [46]. These analyses indicate that tiotropium can be used as an additional controller therapy to ICS in a wide range of patients with asthma. The use of a fixed dose combination of tiotropium with ICS has not been investigated, which may be perceived by some as a disadvantage.

While alternative therapeutic options for paediatric asthma patients are essential, the suitability of the delivery device according to the age and physical capability of the patient is also important to permit accurate dosing and enhance patient adherence. Tiotropium is administered by the Respimat® inhaler, which is a new-generation SMI. This technology delivers medication as a soft mist that reaches both the small and large airways in the lung, thus increasing targeted delivery. The slow-moving mist reduces the reliance on coordination and inspiratory capacity, thereby reducing errors arising from a lack of coordination between actuation and inhalation that can occur with pMDIs, and the need for forceful inhalation as with DPIs. Handling studies have shown that children aged 5 years and older can inhale properly from the Respimat® device. In younger children (patients aged 1–5 years), the use of a valved holding chamber spacer with a facemask is recommended for drug administration [47].

## Conclusion

Management of asthma in children and adolescents is a complex balancing act between diagnosis, treatment, patient education and adherence. Ensuring that patients receive the best available treatment is often not easy due to the paucity of licenced treatments for this age group.

Tiotropium has emerged as a promising new add-on therapy for the management of asthma in patients with partially controlled symptoms of all ages. The recent success of phase III paediatric trials led to the approval of tiotropium for the treatment of asthma in patients aged ≥6 years in the European Union and the United States, therefore improving options for this age group. The positioning of tiotropium in the treatment of asthma in children—either as a treatment escalation after the use of ICS in medium-to-high doses plus LABA, or as an alternative approach to gain better control in children with ICS monotherapy—may be an interesting question for future investigation. Head-to-head studies comparing the combination of either LABAs or tiotropium with ICS would be of interest for treatment decisions, especially for younger children.

## Abbreviations

DPI: Dry powder inhaler
GINA: Global Initiative for Asthma
HCP: Healthcare practitioner
ICS: Inhaled corticosteroids
LABA: Long-acting beta2-adrenoreceptor agonist
LAMA: Long-acting muscarinic antagonist
LTRA: Leukotriene receptor antagonist
pMDI: Pressurised metered-dose inhalers
SABA: Short-acting inhaled beta2-agonists
SMI: Soft mist inhaler
